# Perioperative Optimization Strategies in Major Genitourinary Cancer Surgery

**DOI:** 10.3390/cancers18162588

**Published:** 2026-08-12

**Authors:** Aditi Yellu, Aidan S. Weitzner, Craig Cronin, Joon Kyung Kim, Soum D. Lokeshwar

**Affiliations:** 1 Medical College of Georgia, Augusta University, Augusta, GA 30912, USA; ayellu@augusta.edu; 2 Department of Urology, Johns Hopkins University, Baltimore, MD 21287, USA

**Keywords:** genitourinary malignancies, perioperative optimization, ERAS, prehabilitation, GLP-1 RAs, microbiome, postoperative outcomes

## Abstract

Objective: To evaluate current and emerging perioperative optimization strategies in genitourinary surgery and assess their potential to improve surgical outcomes, particularly in patients with modifiable risk factors. Methods: A literature review was conducted using PubMed databases examining perioperative interventions relevant to patients undergoing surgery for genitourinary cancers. Areas of focus included enhanced recovery after surgery protocols, nutritional optimization, exercise prehabilitation, smoking cessation, glucagon-like peptide-1 receptor agonists, and gut microbiome modulation. Evidence from clinical trials, observational studies, systematic reviews, and current clinical guidelines was reviewed and synthesized. Results: Current evidence supports the use of enhanced recovery after surgery protocols to reduce acute complications, shorten length of stay and decrease recovery time. Nutritional optimization and exercise prehabilitation demonstrated improved functional and metabolic capacity. Smoking cessation improved wound healing and decreased pulmonary and infectious complications. Emerging evidence suggests GLP-1 RAs offer metabolic benefits, and microbiome-targeted interventions may improve postoperative recovery through gut microbiome diversity preservation, although clinical data remains limited. Conclusions: Perioperative strategies demonstrate promising potential to improve outcomes in patients undergoing genitourinary surgeries by addressing modifiable risk factors. Further prospective studies are needed to guide clinical implementation.

## Simple Summary

Patients undergoing surgeries for genitourinary malignancies face risks for complications that can negatively affect recovery, long-term outcomes, and mortality. Those risks are higher in individuals with predisposing conditions such as obesity, diabetes, poor nutritional status, frailty, sarcopenia, and a smoking history. Growing evidence suggests an opportunity for adverse events to be reduced through targeted perioperative strategies. The aim of this review was to examine current and novel approaches to perioperative optimization strategies and highlight their potential role in improving outcomes. We looked into enhanced recovery pathways, nutritional optimization, exercise prehabilitation, smoking cessation, glucagon-like peptide-1 receptor agonists, and gut microbiome modulation as possible interventions and discussed the patient populations most likely to benefit. By identifying modifiable risk factors and implementing these targeted optimization strategies, clinicians can reduce post-operative risks and ultimately improve outcomes for patients undergoing genitourinary surgeries.

## 1. Introduction

Genitourinary (GU) malignancies of the urinary tract and renal parenchyma represent a growing and significant sector of oncologic care. In the United States, bladder cancer accounts for approximately 83,000 cases and over 16,000 deaths annually, while kidney cancer contributes to approximately 81,000 new cases and over 14,000 deaths annually [1]. Globally, GU cancers also contribute substantially to cancer incidence and mortality, with bladder cancer ranking at the 10th most commonly diagnosed cancer worldwide [2]. These malignancies disproportionately affect men and older adults typically exceeding 65 years [1]. The incidence of genitourinary cancers is expected to increase with an aging population and with the continued prevalence of risk factors such as smoking and obesity [2]. Therefore, these cancers represent a remarkable clinical burden, contributing to an increased need for healthcare utilization and resource demand.

Patients with non-muscle-invasive bladder cancer can be managed with endoscopic resections and intravesical therapies. However, patients with muscle-invasive or refractory disease require radical cystectomy, which can be associated with multiple comorbid conditions especially in older patients after surgery [3]. Similarly, upper tract urothelial cancers which arise from the urothelial lining of the collecting system, can be managed endoscopically for low-grade disease. For higher risk patients, a radical nephroureterectomy is typically performed which entails the removal of the kidney, entire ureter, and bladder cuff. This surgery presents unique challenges due to its impact on renal function [4]. For kidney cancer (renal carcinoma), major surgical management remains the primary approach for larger masses with partial nephrectomy for localized tumors and radical nephrectomy for more extensive or difficult to resect disease [5].

For malignancies of the collecting system and renal parenchyma, despite advances in surgical techniques and minimally invasive approaches, the risk of complications remains high [6]. Importantly, these risks are not uniformly distributed, as patients with pre-existing conditions, such as obesity, diabetes, and cardiopulmonary disease, have been shown to experience higher rates of postoperative outcomes following a major GU surgery [7]. Additionally, factors such as frailty and sarcopenia have emerged as strong predictors of postoperative morbidity and mortality [8,9]. While advances in surgical technique and anesthesia have improved technical outcomes [10], complication rates remain high in major GU cancer surgery and are often worse in patients with metabolic and systemic comorbidities.

These postoperative challenges following major GU surgery underscore the critical need for perioperative optimization strategies. As a result, there has been increasing emphasis on perioperative optimization, which are targeted multidisciplinary interventions that are employed across the surgical period to enhance physiologic reserve and reduce surgical risk [11]. Rather than relying on single interventions, modern approaches emphasize using multidisciplinary, multimodal perioperative strategies [12] that involve addressing modifiable risk factors like smoking cessation, nutrition, exercise, and weight management. The objective of this review is to highlight current and novel perioperative strategies to reduce complications and enhance recovery after major GU malignancy surgery.

## 2. Review Methodology

This narrative review was conducted to summarize current evidence regarding perioperative strategies in major GU cancer surgery, including both established interventions and emerging approaches. A literature search was conducted using PubMed, with the final search conducted in June, 2026. Studies published between 2004 and June 2026 were considered. Search terms included combinations of genitourinary cancer, urologic oncology, perioperative optimization, enhanced recovery after surgery (ERAS), nutrition, prehabilitation, exercise, smoking cessation, glucagon-like peptide-1 receptor agonists, GLP-1, microbiome, and probiotics.

Original research articles, randomized control trials, observational studies, systematic reviews, meta-analyses, and relevant clinical guidelines published in English were considered for inclusion. Additional studies were identified through manual review of reference lists. Articles not relevant to perioperative optimization in major GU cancer surgery, conference abstracts without full-text publication, and non-English articles were excluded. No formal risk-of-bias assessment or standardized study quality appraisal was performed because this manuscript was designed as a narrative review. Priority was given to higher-quality evidence most relevant to perioperative optimization in GU surgery.

## 3. Complications of Major Gu Cancer Surgery and Risk Factors

Major GU oncologic surgeries are associated with substantial perioperative morbidity. Radical cystectomy is associated with complication rates up to 64% within 90 days postoperatively, including infectious, gastrointestinal, and cardiopulmonary complications [13,14]. Similarly, patients undergoing radical or partial nephrectomy are at risk for perioperative complications, particularly those with preexisting comorbidities [15].

Although nephrectomy is performed with curative intent, the abrupt reduction in functional nephron mass coupled with perioperative hemodynamic and inflammatory stress frequently precipitate AKI or exacerbate CKD progression [15,16]. Post-nephrectomy AKI is a known predictor of long term CKD risk [17]. The risk of these complications are amplified in patients who have baseline renal impairment, cardiovascular disease, hypertension or other preexisting comorbidities [5]. Over 40% of patients with normal baseline renal function will progress to CKD within 5 years after radical nephrectomy [18]. Nephrectomy is not without other perioperative risks and postoperative complications. These include bleeding, pulmonary and cardiovascular events related to anesthesia, injury to adjacent organs and structures, surgical site infections, wound dehiscence, or postoperative ileus [19,20,21].

Importantly, postoperative complications are not uniformly distributed across patient populations as they are strongly influenced by baseline patient characteristics [13]. Diminished physiologic reserve has been shown to play a central role in determining surgical outcomes. Patients with a poor functional status or physical deconditioning at baseline often exhibit an impaired ability to tolerate surgical stress and reduced cardiopulmonary capacity, which result in worse postoperative outcomes and delayed recovery [22].

Obesity may also lead to worse outcomes after GU cancer surgery. Excess weight and the associated metabolic dysfunction cause a chronic, low-grade inflammatory state and altered renal hemodynamics contributing to poorer perioperative outcomes. Obesity, associated with increased visceral adiposity and excess weight, induces adherent perinephric fat that increases operative difficulty by impairing and lengthening surgical dissection and likely impairing wound healing [23,24]. Excess weight is a known risk factor for RCC and affects the tumor immune microenvironment [25]. Multiple studies have shown an association between elevated BMI and AKI after nephrectomy [16,26,27].

Body weight alone does not entirely measure risk as it does not account for underlying muscle mass. Therefore, sarcopenia, defined as the loss of skeletal muscle mass, has emerged as a powerful predictor of postoperative outcomes. Patients with reduced muscle mass have a limited availability of the amino acids needed for tissue repair and immune response, increasing susceptibility to postoperative infections and delaying recovery [9]. Frailty represents a broader syndrome of systemic vulnerability that encompasses impairments in strength, functional status, and cardiopulmonary reserve. It has been consistently associated with age-independent increased morbidity [8,28].

Metabolic conditions such as diabetes further contribute to perioperative risk through impaired wound healing, immune dysfunction, and increased susceptibility to infection [29]. Similarly, smoking and underlying cardiopulmonary disease reduce cardiovascular and pulmonary reserve, predisposing patients to respiratory and cardiac complications in the postoperative period [30,31]. Collectively, these factors highlight the importance of identifying vulnerable patient populations and underscore the need for targeted perioperative optimization strategies.

## 4. Perioperative Optimization Strategies

Table 1 summarizes the perioperative optimization strategies. Prehabilitation has emerged as a key perioperative optimization strategy, targeting modifiable risk factors to enhance a patient’s physiologic reserve and readiness for the metabolic demands prior to a major surgical intervention. It is a multimodal strategy that consists of exercise training, nutritional support, and psychological support [22].

Exercise-based prehabilitation focuses on improving muscular strength, cardiopulmonary processes, and overall functional capacity prior to surgery. Structured interventions incorporating resistance and aerobic training have been shown to improve a patient’s ability to tolerate surgical stress [22,32]. In a randomized blinded controlled trial, Barberan-Garcia et al. demonstrated that personalized prehabilitation significantly reduced postoperative complications and improved functional capacity in high-risk patients undergoing major abdominal surgery [33]. Similarly, a randomized control trial by Gillis et al. found that prehabilitation improved postoperative functional walking capacity compared to rehabilitation alone following colorectal cancer resection [34]. Meta-analyses have further suggested that prehabilitation may improve postoperative functional recovery and may be associated with reductions in postoperative complications following major intra-abdominal surgery [35]. Although these findings provide a strong rationale for incorporating prehabilitation into GU surgical care, direct GU-specific evidence remains comparatively limited, and the applicability of these results to patients undergoing major GU cancer surgery should therefore be interpreted with caution until validated in prospective GU-specific studies.

Nutritional optimization plays an important role in perioperative care where baseline malnutrition and catabolic stress are common, particularly in patients undergoing major genitourinary surgery. Preoperative nutritional deficits, such as unintentional weight loss and hypoalbuminemia, have been associated with increased postoperative complications and mortality following radical cystectomy [36,37]. Nutritional prehabilitation focuses on correcting these deficits prior to surgery to improve metabolic reserve to support recovery [34]. In addition to generalized nutritional optimization, immunonutrition has emerged as a target strategy to modulate the perioperative inflammatory response. Immunonutrition includes nutritional formulas enriched with arginine, omega-3 fatty acids, and nucleotides that have been shown to reduce postoperative infectious complications and length of hospital stay through the enhancement of immune function and attenuation of inflammatory pathways [38,39]. Although much of this evidence is derived from gastrointestinal oncology, emerging data in GU oncologic patients suggest similar physiologic benefits, including modulation of postoperative inflammatory markers following radical cystectomy [40]. Despite these promising findings, there remains a paucity of high-quality studies defining the role of immunonutrition in improving clinical outcomes in patients undergoing GU oncologic surgeries.

Enhanced Recovery After Surgery (ERAS) is an evidence-based, patient-centered multimodal framework that integrates various perioperative strategies to improve surgical outcomes [41]. In urologic oncology, ERAS protocols for procedures such as radical cystectomy incorporate coordinated interventions across the preoperative, intraoperative, and postoperative phases, some of which include patient education, nutritional optimization, minimized fasting prior to and after surgery, and early postoperative mobilization [42,43]. Additional intra- and postoperative measures, including antibiotic and venous thromboembolism prophylaxis, normothermia maintenance, postoperative nausea and vomiting control, and multimodal analgesia approaches, further contribute to reducing postsurgical complications [42].

Guideline-based ERAS pathways have been associated with a shorter length of stay and faster recovery of bowel function without increasing readmission rates [42,43]. These benefits are thought to arise from the cumulative effect of multiple coordinated interventions that target surgical stress, maintain physiologic homeostasis, and restore function early [12]. Prospective and randomized data in radical cystectomy populations support these findings, demonstrating that standardized recovery protocols can accelerate functional recovery while maintaining safety [44].

Importantly, ERAS is a systems-based perioperative care pathway and should not be viewed as a single intervention. Rather, it serves as a coordinated framework within which prehabilitation strategies, including exercise and nutritional optimization, may be delivered. Recent updates to ERAS pathways for GU surgery recognize the importance of preoperative optimization, with the growing incorporation of prehabilitation strategies such as nutritional support and functional conditioning into the perioperative timeline [43,45]. This reflects a broader shift in GU surgery toward earlier risk modification and proactive patient preparation, particularly in high-risk populations undergoing these complex surgeries.

Smoking cessation represents a critical component of perioperative optimization. In a retrospective propensity-matched cohort study, active smoking has been shown to increase postoperative complications, including pulmonary complications, impaired wound healing, and higher rates of infections, with current smokers demonstrating increased odds of pneumonia, surgical site infections, as well as higher overall mortality risk compared to non-smokers [30,46]. These risks are mediated by impaired tissue oxygenation, reduced microvascular perfusion, and dysregulated immune function, which collectively contribute to delayed healing and increased postoperative morbidity [30].

Preoperative smoking cessation has been shown to significantly reduce postoperative morbidity, with evidence suggesting that even short-term abstinence can improve outcomes, while longer cessation periods can provide a greater benefit [47,48]. Smoking cessation interventions implemented prior to surgery, such as nicotine replacement therapy or counseling, have demonstrated effectiveness in reducing complication risks and increasing quit rates [47]. Furthermore, within the context of ERAS and prehabilitation frameworks, smoking cessation is increasingly recognized as an easily incorporated preoperative optimization strategy. Integrating structured cessation programs into perioperative pathways demonstrates a shift towards proactive risk reduction, particularly in high-risk surgical populations. Taken together, by integrating exercise and nutritional prehabilitation with targeted risk factor modification within standardized ERAS pathways, perioperative care can be optimized to enhance recovery and reduce complications in patients undergoing major GU surgery. However, while several of these interventions are supported by evidence from colorectal and other major abdominal surgical populations, additional GU specific prospective studies are needed to further define their impact on clinical outcomes in urologic oncology.

## 5. Novel and Emerging Optimization Strategies

### 5.1. GLP-1 Receptor Agonists (GLP-1 RA)

#### 5.1.1. Background and Pharmacology of GLP-1 RA

Glucagon-like peptide-1 (GLP-1) receptor agonists (RAs) are a class of medications that have become central to the management of obesity and type 2 diabetes, primarily through appetite suppression and metabolic optimization [49]. These medications mimic the incretin hormone, glucagon-like-Peptide-1, that is secreted by intestinal L-cells in response to nutritional intake [50]. Through the activation of GLP-1 receptors, these medications stimulate glucose-dependent insulin secretion and suppress glucagon release, ultimately resulting in improved glycemic control and weight loss [51]. The weight loss effects of GLP1-RAs are mediated both through central and peripheral mechanisms. Centrally, GLP-1 signaling acts on the hypothalamus and the nucleus tractus solitarius, enhancing serotonergic neuron activity to promote satiety and reduce appetite. Peripherally, they decrease ghrelin levels, improve glycemic control, and delay gastric emptying, further suppressing appetite [49]. These metabolic effects have led to an increasing interest in the use of GLP-1 RAs as an adjunct in perioperative optimization strategies for surgical patients with a history of diabetes, obesity, and metabolic disease [52].

Beyond their metabolic effects, emerging evidence suggests that GLP-1 RAs may possess anti-inflammatory and anti-neoplastic properties [50,53,54]. GLP-1 signaling has been shown to modulate inflammatory cytokine pathways and reduce oxidative stress by improving endothelial function, suggesting a broader role in systemic, metabolic, and inflammatory regulation [50]. Recent oncologic investigations have explored possible associations between GLP-1 signaling pathways and tumor biology, including obesity-associated malignancies and colorectal cancer pathways [53,54,55]. While these findings remain preliminary, they highlight the growing interest in using GLP1-RAs beyond glycemic management.

#### 5.1.2. GLP-1 RAs and Perioperative Optimization

Obesity and diabetes are well-established risk factors for perioperative complications. As a result, metabolic perioperative optimization has become a critical component of care. Given their ability to promote weight loss and improve glycemic control, GLP-1 RAs have emerged as a potential pharmacologic adjunct to prehabilitation, although they are not yet established components within ERAS [49,56].

Weight reduction achieved through GLP-1 RA therapy may be considered to improve surgical candidacy and reduce obesity-related perioperative complications by decreasing visceral adiposity, improving insulin sensitivity, reducing HbA1C, and protecting cells of the cardiovascular and nervous system by reducing inflammation and apoptosis [49,50]. These effects may be particularly relevant in patients undergoing major GU oncologic procedures, given the prevalence of obesity and metabolic dysfunction among patients with renal and bladder malignancies [53,57].

Recent studies have begun evaluating perioperative outcomes associated with GLP-1 RA use, with mixed findings. In a retrospective analysis, Aschen et al. demonstrated that a perioperative GLP-1 RA prescription was associated with significant reductions in risk-adjusted readmission and may be associated with favorable perioperative profiles in diabetic populations [52]. Additionally, Kesaria et al. reported that preoperative GLP-1 RA use was not associated with decreased postoperative complications and mortality following lumbar fusion surgery [58]. A nationwide analysis by Wu et al. has further suggested that preoperative GLP-1 RA therapy may be associated with reduced perioperative cardiorespiratory complications and comparable or improved postoperative outcomes among patients with type 2 diabetes mellitus [59].

#### 5.1.3. Perioperative Safety Such as Aspiration Risk

Despite the potential benefits associated with GLP-1 RAs, there have been initial perioperative safety concerns regarding their effects on gastric motility and the potential risk of pulmonary aspiration during anesthesia [60,61]. GLP-1 RAs delay gastric emptying through modulation of gastric motility and vagal pathways, resulting in prolonged retention of gastric contents even after standard preoperative fasting periods [49,51]. The presence of these gastric contents during the induction of anesthesia may increase the risk of regurgitation and pulmonary aspiration [60].

The perioperative concerns surrounding GLP-1 RAs were prompted by case reports describing retained gastric contents and pulmonary aspiration events in patients taking semaglutide and undergoing anesthesia despite adherence to standard fasting guidelines [62,63,64]. In response to these reports, the American Society of Anesthesiologists (ASA) released consensus-based guidance in 2023 recommending temporary discontinuation of GLP-1 RAs prior to elective procedures, including holding daily formulations on the day of surgery and weekly formulations for one week preoperatively [60]. These recommendations were largely precautionary, as they were developed in the setting of limited perioperative evidence and evolving safety data.

However, more recent evidence suggests the perioperative risks associated with GLP-1 RAs may be lower than initially anticipated. Notably, a large retrospective cohort study by Dixit et al. found no significant increase in postoperative respiratory complications among patients receiving GLP-1 RAs, including those undergoing urgent and emergent procedures who were unable to discontinue therapy before surgery [61]. Likewise, Wu et al. found no associated risks in perioperative cardiorespiratory complications and mortality in a nationwide propensity-score matched cohort of patients with type 2 diabetes [59]. Additional retrospective cohort analyses by Aschen et al. further reported that perioperative GLP-1 RA use was not associated with worse perioperative outcomes, but rather favorable ones in selected populations [52]. These findings have contributed to a shift away from the universal discontinuation strategies towards more individualized perioperative management approaches.

Current perioperative management approaches emphasize individualized risk assessment and collaborative decision-making regarding continuation or temporary discontinuation of GLP-1 receptor agonist therapy [65]. American Society of Anesthesiologists consensus guidance initially recommended withholding daily GLP-1 RAs on the day of surgery and weekly formulations for one week prior to elective procedures due to concerns regarding delayed gastric emptying and aspiration risk [60]. However, more recent multi-society clinical practice guidance supports continuation of these agents for most patients while recommending individualized assessment based on gastrointestinal symptoms, the dose-escalation phase, aspiration risk, and the planned surgical procedure [65]. Patients at higher risk for delayed gastric emptying may require additional precautions such as preoperative liquid diets or gastric ultrasounds to assess aspiration risk [65]. At the same time, discontinuation of GLP-1 RAs must be balanced against the risks of worsening glycemic control and the patient’s metabolic needs [65]. Following surgery, patients should work closely with the prescribing physician to determine the appropriate timing for resuming therapy and whether dose re-escalation is necessary.

#### 5.1.4. Emerging Evidence of GLP-1 RAs in Urologic Surgery

Interest in GLP-1 RAs within urology has expanded considerably in recent years, extending beyond their traditional roles in diabetes management and weight reduction. GLP-1 RAs may also provide anti-inflammatory and potential oncologic benefits in genitourinary oncology, although the available clinical evidence remains limited and is largely derived from prostate cancer studies [66].

Emerging evidence suggests that GLP-1 RA therapy prior to a partial nephrectomy may be associated with reduced perioperative complications such as AKI, pneumonia, readmission and ileus, arrhythmias, blood transfusion requirements, and hospitalization, all of which contribute to an improved overall survival [67]. Additionally, Banihashem Ahmad et al. demonstrated an association between GLP-1 RA therapy and improved renal function in a propensity-matched cohort analysis, associated with decreased progression to advanced kidney disease (CKD) and dialysis dependence [68]. A pilot retrospective cohort study utilizing the TriNetX database by Weitzner et al. evaluated perioperative and renal outcomes following nephrectomy in patients receiving GLP-1 RA therapy [69]. Given ongoing concerns regarding delayed gastric emptying and perioperative safety, this study reported no significant increase in perioperative morbidity or adverse renal outcomes, suggesting that these medications may be considered for incorporation into perioperative management for selected nephrectomy patients [69]. Collectively, these emerging findings suggest that GLP-1 receptor agonists may represent a promising adjunct in perioperative metabolic optimization for urologic surgery patients, though further prospective and urology-specific studies are needed to better define their effects.

### 5.2. Gut Microbiome and Perioperative Optimization

The gut microbiome has emerged as an increasingly important area of interest in perioperative medicine due to its role in immune regulation, inflammatory signaling, and metabolic homeostasis [70]. Recovery following a major surgery can be complicated by postoperative outcomes, anastomotic leak, GI dysmotility, and cancer recurrence, in which the implication of the gut microbiome has started to emerge [71,72,73]. Gut microbial balance may already be disrupted preoperatively due to underlying malignancy, preoperative fasting, and antibiotic therapy, which can significantly disrupt microbial homeostasis and epithelial repair [71,73]. Alterations to the microbiome can impair postoperative recovery and lead to complications following major GI surgery, such as surgical site infections and impaired healing [73,74].

Microbiome-related perioperative measures may be particularly relevant in patients undergoing radical cystectomy and nephrectomy, where urinary diversion, perioperative fasting, antibiotic exposure, and dietary alterations may significantly disrupt gut homeostasis [75,76]. Emerging evidence has linked the gut microbiome to oncologic outcomes and immunotherapy responses in GU malignancies, particularly RCC and bladder cancer [75,77]. In metastatic RCC, dysbiosis could alter responsiveness to immune checkpoint inhibitor therapy, with differences in microbiome associated with variability in treatment efficacy and clinical outcomes among patients receiving immunotherapy [77]. Additionally, obesity and metabolic syndromes, which are common among patients undergoing these surgeries, have been closely associated with lower diversity, leading to immune dysregulation [78]. These findings highlight the potential relevance of microbiome modulation as a perioperative optimization for GU malignancies.

Potential microbiome-targeted perioperative interventions currently under investigation include probiotics, prebiotics, synbiotics, and fecal microbiota transplantation (FMT) [79]. Several studies have demonstrated that perioperative microbiome therapy may reduce postoperative complications such as infections and improve recovery following major surgery [80,81,82]. In a randomized control trial, Liu et al. demonstrated that patients undergoing colorectal surgery who had taken probiotics preoperatively had improved integrity of gut mucosal barrier by benefiting the fecal microbiota, and decreased infectious complications [80]. Similarly, in another randomized controlled trial, Kotzampassi et al. showed that probiotic formulation as prophylaxis decreased postoperative complications in colorectal surgery patients, namely mechanical ventilation, infections, and anastomotic leakage [81].

**Figure 1:**
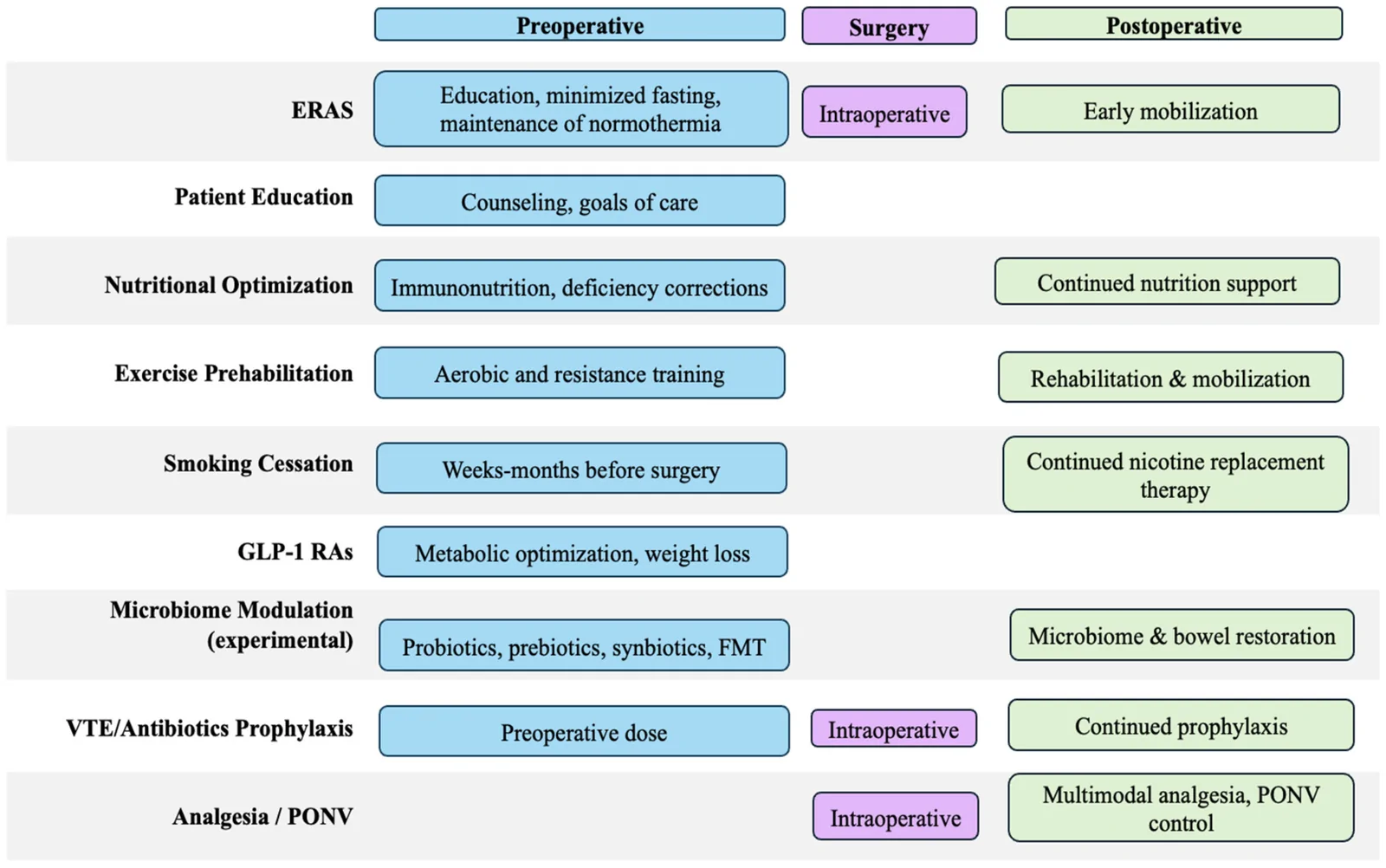
Timeline of Established and Emerging Perioperative Strategies for Patients Undergoing GU Surgeries.

Beyond the perioperative setting, oncology-focused interventions have also explored microbiome modulation as a strategy to improve immunotherapy response. Although these studies do not directly evaluate perioperative optimization in GU surgery, they provide mechanistic evidence supporting the potential role of the gut microbiome in cancer treatment. In a randomized clinical trial involving patients with metastatic renal cell carcinoma, Dizman et al. found the probiotic formulation CBM588 in combination with nivolumab and ipilimumab was associated with improved clinical outcomes [83]. In studies involving patients with advanced melanoma and those receiving anti-PD-1 therapy, fecal microbiota transplantation (FMT) was associated with favorable changes in immune cell infiltration, gene expression profiles in both the gut lamina propria and the tumor microenvironment, and with some patients demonstrating improved responsiveness and reversal of resistance to immunotherapy [84,85]. Although evidence of these perioperative strategies remains limited in the context of GU surgeries, these findings support the growing potential of microbiome-targeted therapies as investigational approaches for perioperative optimization. However, additional GU-specific prospective studies are needed before their routine implementation can be recommended. Figure 1 provides a summary of these collective strategies.

## 6. Conclusions

As the incidence of GU malignancies continues to rise, optimizing perioperative care prior to surgery is imperative. Patients with modifiable risk factors tend to face an increased risk of surgical complications, highlighting the importance of targeted optimization strategies. Prehabilitation approaches such as exercise, nutritional optimization, and smoking cessation, together with comprehensive ERAS pathways, can enhance recovery and reduce postoperative complications. Emerging interventions, including GLP-RAs and microbiome-targeted therapies, offer promising avenues in select patient populations by improving perioperative outcomes. Much of the current evidence remains preliminary, especially within GU populations, emphasizing the need for further randomized controlled studies evaluating these novel strategies in patients undergoing GU surgeries. Continued refinement and integration of various perioperative optimization strategies have the potential to greatly improve recovery, minimize complications, and ultimately enhance the outcomes for patients with GU malignancies. As perioperative care continues to improve, a personalized, multimodal approach is essential in providing high-quality surgical and oncologic care for patients with GU malignancies.

## Figures and Tables

**Table 1:** Summary of Perioperative Optimization Strategies for GU Surgeries.

Optimization Strategy	Patients Most Likely to Benefit	Key Interventions	Proposed Benefits	Initiation Time	Patient Selection	Level of Evidence	Contraindications/Considerations
ERAS	All patients undergoing GU surgery	Preoperative education, nutritional optimization, minimized fasting, VTE + antibiotic prophylaxis, multimodal analgesia, early mobilization, normathemia, postoperative nausea and vomiting control	Reduced complications, shorter LOS, faster recovery	Throughout perioperative period	All	High (RCTs, guidelines)	None
Nutritional Optimization	Malnourished patients, patients with unintentional weight loss (cancer cachexia), or hypoalbuminemia	Immunonutrition, corrections of deficiencies	Improved metabolic reserve, reduced infections complications, reduced LOS, enhanced immune health via inflammatory modulation	As early as possible before surgery	Patients with malnutrition, weight loss, sarcopenia, or hypoalbuminemia	Moderate (observational studies, guidelines)	None
Exercise Prehabilitation	Frail, deconditioned, sarcopenic, or elderly patients	Resistance training, aerobic training, postoperative walking	Improved functional capacity, ability to tolerate surgical stress, and physiologic reserve	As early as possible before surgery (ideally ≥4 weeks)	Patients with frailty, sarcopenia, or reduced functional capacity	High (RCTs, meta-analyses)	None; individualized based on functional status
Smoking Cessation	Current smokers or recent tobacco user	Structured cessation programs, behavioral counseling, nicotine replacement therapy	Decreased pulmonary complications, improved wound healing, decreased infectious complications (pneumonia, surgical site infections), decreased mortality	As early as possible before surgery	Current smokers	High (RCTs, guidelines)	None
GLP-1 RAs	Obese patients, metabolic syndrome, or diabetes	Semaglutide (Ozempic), Liraglutide, and Dulaglutide	Weight loss, improved glycemic control, decreased inflammation and potential reduction in neoplasms	≥4 weeks before surgery, as directed by prescribing clinician	Patients with obesity and/or type 2 diabetes who are appropriate candidates for GLP-1 RA therapy	Emerging (retrospective studies, database analyses)	Individualized perioperative management; higher-risk patients may require temporary discontinuation
Microbiome Modulation	Patients at high risk for microbiome disruption, including those undergoing bowel diversion, receiving perioperative antibiotics, undergoing immunotherapy, and individuals with obesity or metabolic syndrome	Experimental: Probiotics, prebiotics, synbiotics, and FMT	Reduced infectious complications, improved postoperative recovery, preservation of gut microbial diversity, and potential enhancement of immunotherapy response	Perioperative period	Patients at high risk for microbiome disruption	Experimental (non-GU evidence, preclinical and early clinical studies)	None established; investigational

## Data Availability

No new data were created or analyzed in this study. Data sharing is not applicable to this article.
